# Editorial: Cardiovascular anthropometry for large scale population studies

**DOI:** 10.3389/fcvm.2023.1353557

**Published:** 2024-01-09

**Authors:** Basil Nwaneri Okeahialam, Okechukwu Samuel Ogah

**Affiliations:** ^1^ University of Jos, Jos, Nigeria; ^2^ Cardiology Unit, Department of Medicine, Jos University Teaching Hospital, Jos, Nigeria; ^3^ Cardiology Unit, Department of Medicine, University of Ibadan, Ibadan, Nigeria; ^4^ Cardiology Unit, Department of Medicine, University College Hospital, Ibadan, Nigeria

**Keywords:** anthropometry, cardiovascular diseases, abdominal height, body mass index, population studies

**Editorial on the Research Topic** Cardiovascular anthropometry for large scale population studies

Obesity and overweight are associated with development of cardiovascular diseases (CVD) such as hypertension, diabetes mellitus (DM), and the metabolic syndrome, as well as clinical conditions resulting from the consequent atherosclerotic cardiovascular diseases like stroke, heart attacks and peripheral artery disease. According to the World Health Organization (WHO), obesity, and overweight refer to abnormal or excessive fat accumulating in the body, which in turn impact negatively on health. This realization that overweight and obesity have an adverse effect on health has been recognized as far back as the 6th century BC. The WHO therefore came up with the body mass index (BMI) as measure of overweight and obesity for use in epidemiological studies.

This issue of Frontiers in Cardiovascular Medicine is dedicated to articles in Cardiovascular anthropometry and simple affordable non-anthropometric measures that tend to refine the age long WHO recommended measure, BMI making it more predictive of cardiometabolic diseases.

Whereas Agbo et al. (1) came up with a new index called the Abdominal Height which they recommended for wide scale use in sub-Saharan Africa, several other workers have, utilizing existing anthropometric measures come up with indices predictive of cardiovascular disease risk.

One is the weight adjusted waist circumference index, a quotient of waist circumference and square root of weight. It is a simple surrogate for fat mass accumulation. Applying it to the NHANES data of 1999–2018, Zhang et al. were able to show a linear and significant association with prevalence of heart failure. This has been known and as stated by Koparkar and Biswas (2) cardiomyopathy results as a direct consequence of fat in what has been called adipositas cordis.

Vascular calcification, a measure of severity of atherosclerotic cardiovascular disease is becoming of high utility in predicting higher cardiovascular disease burden. This is most evident with coronary artery disease (3). In this issue, Li et al. used an anthropometric index a body surface index (ABSI) derived from height body mass index and weight circumference to interrogate abdominal aortic calcification (AAC). They showed that ABSI correlated positively with AAC and that its discriminant ability superseded those of the different anthropometric indices from which it was derived.

The body roundedness index (BRI) is another novel anthropometric index that predicts body fat distribution better than BMI. It has been shown to better predict metabolic syndrome and cardiometabolic diseases than other common anthropometric indices (4) as it predicts both body fat and visceral adiposity. In this issue, Ding et al. applying this index in a Chinese middle age to elderly population showed that BRI in the higher trajectory was significantly related to all cause and cardiovascular mortality.

Atrial fibrillation (AF), the commonest arrhythmia in clinical practice is known to be a cause of increased all cause and cardiovascular mortality in the population. It has been shown to be related to body fat (5). From anthropometry, blood chemistry and some life style factors Woo et al. derived more precise cardiovascular disease indices: predicted body fat mass index and predicted lean body mass index. They found that the risk of AF rises with these indices except in those underweight by the BMI classification.

Non-alcoholic fatty disease is known to be a manifestation of metabolic syndrome, a forerunner to cardiometabolic diseases (6). Fatty Liver Index (FLI) derived from certain anthropometric and blood chemistry measures was used by Niu et al. to detect ischemic heart disease (IHD) using data from the 1999–2016 NHANES. This study published in this issue showed a linear and positive relationship between FLI and prevalent IHD.

Using R analysis Li et al. in this issue tried to determine risk factors for hypertension in an overweight and obese population. Seven such factors were identified but age and uric acid exhibited synergistic interaction making them a potential reference standard for initiation of preventive and curative action in overweight and obese hypertensives.

Weight management has been stressed as key in preventing cardiovascular diseases in diabetics (7). Li et al. in this issue interrogating a population of Chinese diabetics found that the burden of cardiovascular disease risk factors changed for the better with weight reduction. This means that for reduction in cardiovascular disease morbidity in diabetics, every effort must be made to normalize body weight.

Quality of diet has a bearing on prevalent cardiovascular diseases (8), hence the impact of dietary modification in the management of cardiovascular diseases. Mohamadi et al. in this issue utilized the diet quality index (DQI) to study overweight and obese women for cardiometabolic risk factors. They were able to show a relationship giving fillip to the need for good quality diet in prevention of cardiometabolic diseases.

Finally Peng et al. using NHANES 1999–2018 data looked at weight change and predicted 10 year risk for atherosclerotic vascular disease in an American population of the elderly. They were able to show that stable weight rather than flux in weight was more beneficial for maintaining cardiovascular health

On the whole, the various contributions in this issue show that though relevant BMI is not very precise in predicting risk of cardiometabolic diseases and new paradigms tracking fat mass and fat location in the body rather than overall weight which includes bone and muscle would be the way to go. This is where the novel anthropometric index, Abdominal Height stands out. It does not require more anthropometric measures for inclusion into an equation to derive, neither does it involve blood sampling and biochemical analyses which introduce inconvenience and cost.
